# Antihistaminic effect of *Bauhinia racemosa* leaves

**DOI:** 10.4103/0975-1483.80301

**Published:** 2011

**Authors:** SA Nirmal, RB Laware, R A Rathi, VV Dhasade, BS Kuchekar

**Affiliations:** *Department of Pharmacognosy, Pravara Rural College of Pharmacy, Loni, India*; 1 *Department of Pharmaceutics, Pravara Rural College of Pharmacy, Loni, India*; 2 *Department of Pharmaceutical Chemistry, MAEER’s Maharashtra Institute of Pharmacy, Kothrud, Pune, Maharashtra, India*

**Keywords:** Antihistaminic, asthma, *Bauhinia racemosa*, catalepsy, clonidine

## Abstract

*Bauhinia racemosa* Lam. (Caesalpiniaceae) leaves have been used in the treatment of asthma traditionally and we therefore undertook this study to scientifically validate its benefit in asthma using suitable animal models. Antihistaminic principles are known to be useful in the treatment of asthma; hence, in the present work, the antihistaminic activity of an ethanol extract of *B. racemosa* (at a dose of 50 mg/kg, i.p.) was assessed using clonidine-induced catalepsy and haloperidol-induced catalepsy in Swiss albino mice. The results showed that the ethanol extract inhibits clonidine-induced catalepsy but there is no effect on haloperidol-induced catalepsy. This suggests that the inhibition is through an antihistaminic action and that there is no role of dopamine. Hence, we concluded that the ethanol extract has significant antihistaminic activity. The polar constituents in the ethanol extract of leaves of *B. racemosa* may be responsible for the antihistaminic activity and *B. racemosa* may therefore have a role in the treatment of asthma.

## INTRODUCTION

*Bauhinia racemosa* Lam. (Caesalpiniaceae) is a small bushy tree with drooping branches. The leaves are green and broader than long. The flowers are white or pale yellow, terminal or leaf-opposed racemes.[1–3] A new tetracyclic lupeol, betulin, β-sitosterol, and tetracyclic 2, 2-dimethyl chroman have been isolated from the roots.[4,5] The seed contains flavonoids, crude protein, and lipid.[6,7] A methanolic extract of the stem and bark are used as an anti-inflammatory, analgesic and antipyretic.[8] A methanolic extract of the flower buds is used in the treatment of peptic ulcer.[9] The whole plant is used as a veterinary medicine in central India. The present work was undertaken to evaluate the traditionally recognized antiasthmatic property of the leaves of B. *racemosa*.

Catalepsy is a condition in which the animal maintains an imposed posture for a long time before regaining its normal posture. Catalepsy is a sign of the extra-pyramidal effect of drugs that inhibit dopaminergic transmission or increase histamine release in brain. Clonidine, a α_2_ -adrenoceptor agonist, induces dose-dependent catalepsy in mice, which is inhibited by histamine H _1_ -receptor antagonists but not by H _2_ -receptor antagonists.[10]

In the present work we have attempted to evaluate the antihistaminic activity of the plant in order to assess if there is a basis for its traditional use in asthma.

## MATERIALS AND METHODS

### Plant material

The plant B *racemosa* was collected from Ahmednagar district (Maharashtra) in November, 2007, and authenticated by the Botanical Survey of India, Pune (Voucher specimen No. SCDBR1).

### Animals

Male albino mice (Swiss strain) weighing 22-25 g were housed under standard laboratory conditions in groups of six each. The animals had free access to food and water. The ethics committee of the institute approved the protocol of the study.

### Drugs and chemicals

The drugs used were: clonidine (Unichem, India), haloperidol (Sunpharma, India.), and pheniramine maleate (Pfizer Ltd.); all were purchased from a commercial source. Chemicals used were: ethanol AR (PCL, India) and Tween 80 AR (PCL, India).

### Preparation of extract

The fresh leaves of B *racemosa* were shade-dried, crushed to produce a coarse powder, and subjected to extraction in a Soxhlet extractor using ethanol. The extract was filtered while hot and concentrated by vacuum distillation; it was then dried in open air to give a yield of 15.3% w/w.

### Anticataleptic activity

Effect on clonidine-induced catalepsy: The bar test was used to study the effect of various extracts on clonidine-induced catalepsy.[11] Clonidine (1 mg/kg, s.c.) was injected into mice (n=6) pretreated 30 min before with vehicle (Tween 80 in distilled water; 5 mL/kg, p.o.) and ethanol extract (50 mg/kg, i.p.) or standard drug pheniramine maleate (10 mg/kg, i.p.) The dosages were selected based on an acute toxicity study (data not shown). The forepaws of mice were placed on a horizontal bar (1 cm in diameter, 3 cm above the table) and the time taken for the mouse to remove its paws from the bar was noted for each animal; the duration of catalepsy was measured at 0, 15, 30, 60, 90, 120, 150, and 180 min.

*Effect on haloperidol-induced catalepsy:* The same procedure was followed using haloperidol as the inducing agent.[11] Haloperidol (1 mg/kg, i.p.) was injected to mice (*n*=6) pretreated 30 min before with vehicle (Tween 80 in distilled water; 5 mL/kg, p.o.) or ethanol extract (50 mg/kg, i.p.). The duration of catalepsy was measured at 0, 15, 30, 60, 90, 120, 150, and 180 min.

### Statistical analysis

The data are presentedas mean ± SEM. The data was analyzed by one-way ANOVA followed by Dunnet’s test. Prism Graph Pad 3 was used for statistical analysis. P<0.05 was considered significant.

## RESULTS

**Figure 1:**
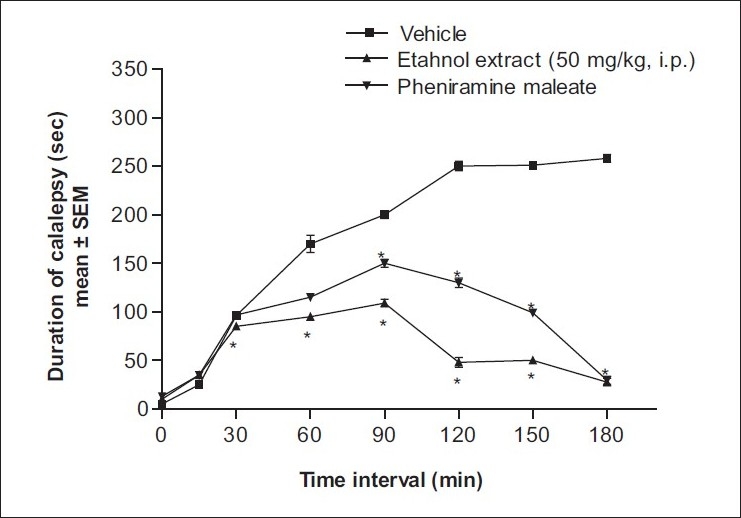
Effect of ethanol extract of *B. racemosa* on clonidine-induced catalepsy in mice. *P<.05 compared to vehicle-treated group (one-way ANOVA followed by Dunnett’s test)

**Figure 2:**
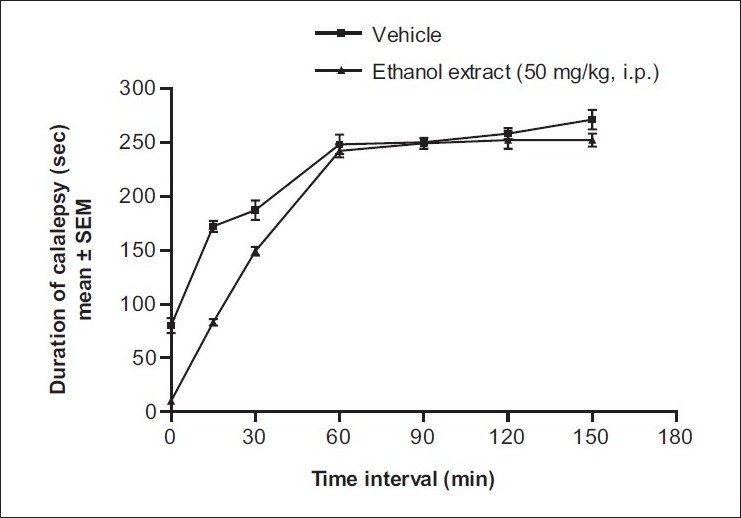
Effect of various extract of *B. racemosa* on haloperidol-induced catalepsy in mice

The results show thattheethanol extract of leaves of B *racemosa* (50 mg/kg, i.p.) inhibits clonidine-inducedcatalepsy [Figure 1] but not haloperidol-induced catalepsy [Figure 2]. The inhibition of catalepsy was comparable with that with standard drug pheniramine maleate.

## DISCUSSION

Several drugs are known to induce catalepsy in animals. Chopra and Dandiya have studied the relative role of acetylcholine and histamine in perphenazine-induced catalepsy and suggested that the anticholinergic activity of antidepressants might be due to an increase in the dopamine content in brain or because of their ability to inhibit the release of acetylcholine.[12] The authors also showed that different stages of catalepsy appear to be directly correlated with brain histamine content. Uvnas studied mast cell degranulation and its correlation with the release of histamine after administration of a mast cell degranulating agent (Compound 48/80).[13] Lakdawala *et al*. have shown that clonidine releases histamine from mast cells in a similar manner to a selective liberator like compound 48/80.[14] They also showed that pretreatment with L-histidine, a precursor of histamine, potentiated clonidine-induced catalepsy in a dose-dependent manner. Muley *et al*. showed that intracerebroventricular injection of histamine in conscious mice induced catalepsy, which was inhibited by H_1-_ receptor antagonist but not by H_2_ -receptor antagonist.[15] It is known that clonidine releases histamine from mast cells.[14] Schwatz identified histamine-containing mast cells in the brain.[16] Clonidine-induced release of histamine from mast cells is inhibited by the α_2_ -adrenoceptor blocker, prazosin.[17] Neuroleptic agents also induce catalepsy, but by a different mechanism: they inhibit dopamineD_2_ -receptors in substantia nigra.[18,19]

The findings of this study indicate that the ethanol extract of leaves of *B. racemosa* can inhibit clonidine-induced catalepsy but not haloperidol-induced catalepsy. From this study we can conclude that the cataleptic effect of clonidine in the mouse is mediated by histamine release from mast cells. The effect of this extract on clonidine-induced catalepsy is probably due to its mast cell-stabilizing property. The plant does not have activity on dopaminergic transmission. Thus the polar constituents may be useful as an antihistaminic and be used in the treatment of asthma.
